# Secular Trends in Height, Body Mass, and BMI Among Polish Boys in Eastern Regions from 1986 to 2021: Cross-Decade Analysis of Nutritional Status

**DOI:** 10.3390/jcm14165767

**Published:** 2025-08-14

**Authors:** Agnieszka Wasiluk, Jerzy Saczuk

**Affiliations:** Department of Health Promotion, Faculty of Physical Education and Health in Biala Podlaska, Jozef Pilsudski University of Physical Education in Warsaw, 00-968 Warsaw, Poland; jerzy.saczuk@awf.edu.pl

**Keywords:** secular trend, physical development, boys, underweight, overweight, obesity

## Abstract

**Background/Objectives:** Secular trends in children’s physical development are important indicators of population health, nutritional status, and socioeconomic conditions. This study aimed to assess long-term changes in the height, weight, Body Mass Index (BMI), and nutritional status of boys from Eastern Poland between 1986 and 2021. **Methods:** Anthropometric data were collected from 13,172 boys aged 8, 13, and 17 years at five time points (1986, 1996, 2006, 2016, and 2021). Standardized measurement protocols were used throughout the study. The BMI was calculated and categorized using international cut-off points for age and gender. Secular changes in the height, weight, and Body Mass Index (BMI) were analyzed using an analysis of variance (ANOVA) with post hoc tests, and differences in dietary categories were assessed using chi-square tests (*p* ≤ 0.05). **Results:** The height, weight, and BMI increased significantly across all ages. The largest height gain was seen in 13-year-olds, while the greatest BMI increase occurred between 2016 and 2021. The overweight and obesity prevalence rose sharply by an average of 21.70% across age groups, with the normal BMI prevalence decreasing by 18.41%. The underweight prevalence declined, especially among adolescents; however, this likely reflects a general upward shift in the BMI rather than a true nutritional improvement. **Conclusions:** Strong secular trends are evident, influenced by global and local socioeconomic factors, including Poland’s EU accession and the COVID-19 pandemic. While an increased height suggests better living standards, the rising overweight and obesity rates indicate emerging health risks. Due to the lack of direct lifestyle and socioeconomic data, further research incorporating these factors and the pubertal BMI variability is needed to clarify underlying causes. Targeted regional strategies promoting healthy diets, physical activity, and lifestyles are urgently required.

## 1. Introduction

Tracking long-term changes in somatic characteristics such as body weight and height serves as a sensitive indicator of a population’s general health, living conditions, food availability, and quality of healthcare [1]. In the 20th and 21st centuries, many countries have reported significant increases in the average height of children and adolescents, often interpreted as a positive sign of improved living and health conditions [2,3]. However, changes in body weight have shown more variation, frequently resulting in imbalanced weight-for-height proportions and an increased risk of overweight, obesity, or underweight. Therefore, analyzing long-term trends in both height and weight is essential for understanding broader health patterns in child and adolescent populations.

While secular trends in basic anthropometric traits are well documented, trends in the Body Mass Index (BMI) are equally critical. In recent decades, the nutritional status of children and adolescents has become a major focus of global research. The increasing prevalence of both an excessive and insufficient body weight among growing individuals contributes to the complex and increasingly recognized phenomenon of the double burden of malnutrition, which has lifelong health consequences [4,5]. According to recent data from the NCD Risk Factor Collaboration [6], covering the years 1990–2022, the combined prevalence of underweight and overweight in children and adolescents has increased in 69–70% of countries worldwide, with rising obesity being the primary contributor. Still, underweight—an indicator of undernutrition—remains a significant issue in many nations, particularly in South Asia and parts of Africa. In 2022, obesity was more prevalent than underweight among girls in 67% of the analyzed countries and among boys in 63%; the reverse was true in only 18% and 21% of countries, respectively. These global patterns show a wide variation in the local intensity and dynamics, depending on the socioeconomic and cultural context.

Poland, particularly its eastern regions, has also seen the coexistence of undernutrition and excess body weight, which may reflect varying rates of socioeconomic transformation [7]. Recent national surveillance data confirm that overweight and obesity affect approximately 30–33% of children aged 8–15, with the highest prevalence among younger age groups. According to the 2021 COSI study, 33% of 8-year-olds in Poland had excess body weight, including 15% classified as obese [8]. Studies by Saczuk [9], conducted between 1986 and 2016, indicate that both underweight and overweight are most prevalent among younger children, with slightly lower rates in older age groups. Over the past two decades, Poland has experienced profound socioeconomic change, especially following its accession to the European Union. These transformations have contributed to regional health disparities. While the eastern voivodeships report childhood overweight and obesity rates exceeding 35%, lower rates are observed in more urbanized regions, such as Pomorskie (26%), Mazowieckie (28%), and Dolnośląskie (27%) [10].

These shifts have contributed to growing regional disparities in living standards and child health [11,12,13,14]. Similar long-term trends have been observed among physical education students, suggesting the continuity of secular processes in physical development across life stages [15]. The last five years, in particular, have brought additional challenges due to the implementation of the “Family 500+” welfare program and physical activity restrictions during the COVID-19 pandemic [16,17,18]. Notably, the 2021 wave of the COSI study—conducted shortly after pandemic-related lockdowns—documented a marked increase in overweight and obesity among school-aged children compared to previous years, likely reflecting reduced physical activity and changes in dietary habits [8].

In this context, monitoring secular trends in somatic development and nutritional indicators among children and adolescents is especially important in regions with lower socioeconomic development. The aim of this study was to assess long-term changes in height, weight, BMI, and the prevalence of the underweight, overweight, and healthy weight status among boys living in Eastern Poland between 1986 and 2021.

## 2. Materials and Methods

In 1986, as part of Key Problem 10.7, a study was conducted involving children and adolescents aged 7 to 18 years residing in the former eastern voivodeships of Poland (Suwalskie, Białostockie, Bialskopodlaskie, Chełmskie, Zamojskie, Przemyskie, and Krośnieńskie). These areas comprised the eastern borderland region of Poland. Following the administrative reform of 1998, these territories now constitute the eastern parts of the Podkarpackie, Lubelskie, Podlaskie, Warmińsko-Mazurskie, and, to a lesser extent, the Mazowieckie voivodeships. Measurements in these regions were repeated in 1996, 2006, 2016, and again in 2021 as part of statutory research conducted by the Józef Piłsudski University of Physical Education in Warsaw (projects D.S. 49 and D.S. 203). With the permission of the project’s principal coordinator, the present study also includes data from 182 boys assessed in 2021 under the “Active Return to School–PE with the University of Physical Education” program, conducted in the Podlaskie, Lubelskie, and Podkarpackie voivodeships. The study included boys aged 7 to 18 years residing in the designated regions who had complete anthropometric data. Participants outside the defined age ranges for each group or with missing key measurements were excluded from the analyses.

All measurements across the various waves of observation were carried out using a consistent research methodology. This study utilizes data from a total of 13,172 boys, categorized into three age groups corresponding to successive stages of biological development: prepubertal (8 years old, *n* = 5190), pubertal (13 years old, *n* = 4973), and postpubertal (17 years old, *n* = 3009). A detailed distribution of the study sample by chronological age and year of observation is presented in Table 1.

This study was conducted in accordance with the principles of the Declaration of Helsinki and was approved by the Senate Ethics Committee of the Józef Piłsudski University of Physical Education in Warsaw.

From the questionnaire data, information regarding the participants’ dates of birth was utilized. Chronological age was calculated in decimal format as the difference between the date of measurement (survey) and the date of birth, accurate to two decimal places of a calendar year. Age was then recoded into whole-year categories according to the following criterion: the 8-year-old group included boys aged between 7.50 and 8.49 years. The same procedure was applied to the other age groups.

Anthropometric measurements were carried out in accordance with established anthropometric protocols [19]. Measurements included body height and body mass, which were subsequently used to calculate Body Mass Index (BMI), defined as the ratio of body mass in kilograms to the square of height in meters.

Within each chronological age group, arithmetic means and measures of variability were calculated for body height, body mass, and BMI. Statistical analyses were conducted using data from the years 1986, 1996, 2006, 2016, and 2021. To assess secular changes, differences between the values of individual somatic indicators across successive time points were computed. These results enabled the identification of developmental trends in the studied parameters.

The statistical significance of differences between groups was tested using analysis of variance (ANOVA) followed by the Newman–Keuls post hoc test, with the significance level set at *p* ≤ 0.05. All reported anthropometric values were rounded to two decimal places for clarity and consistency in data presentation. No additional standardization or smoothing of data was applied.

Additionally, the sample was classified into groups of underweight, normal weight, and overweight based on BMI. Classification criteria were based on the international BMI cut-off points developed by Cole [20] and Cole et al. [21]. This allowed for the calculation of the percentage of boys classified as underweight (including grades I°, II°, and III°), as having normal BMI, and as overweight (including overweight and obese categories). These calculations were performed separately for each chronological age group and across each time point of observation, which allowed for the identification of both the magnitude and direction of changes in BMI distribution. The statistical significance of differences in group proportions was assessed using the chi-square (χ^2^) test, with the significance level set at *p* ≤ 0.05. Data from 1996 were excluded from the prevalence calculations due to the lack of access to archived individual-level data, which made reliable BMI classification impossible for that year. Given that sample sizes in the other years were sufficiently large and relatively balanced, no weighting or adjustment for unequal sample sizes was applied. Preliminary analyses confirmed similar demographic and anthropometric characteristics across study waves, minimizing potential bias. Therefore, the exclusion of 1996 data and absence of weighting do not compromise the validity of the observed secular trends.

## 3. Results

**Figure 1:**
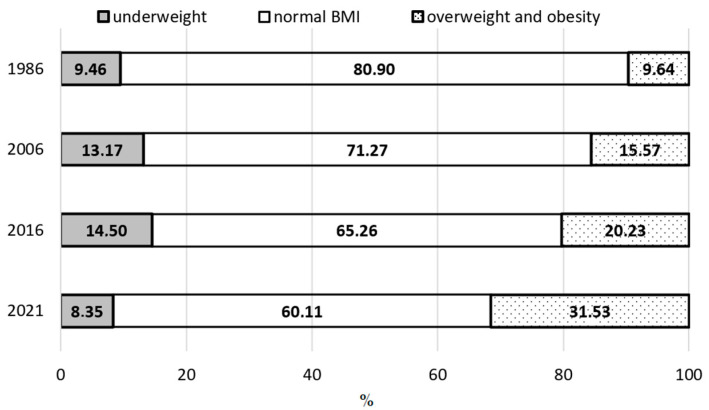
Prevalence of underweight, normal weight, and excess body weight among boys aged 8 years in 1986, 2006, 2016, and 2021.

Between 1986 and 2021, a systematic increase in the mean body height was observed among boys from the eastern regions of Poland (Table 2, Figure 1). The most pronounced changes occurred in the group of 13-year-olds, with an increase of 10.62 cm, followed by 8-year-olds (7.16 cm) and 17-year-olds (4.83 cm). An analysis of long-term trends across the study periods revealed that, within the prepubertal group, the most significant increase in body height occurred between 1996 and 2006 (2.86 cm). In the pubertal (4.78 cm) and postpubertal (2.61 cm) groups, the most substantial changes were recorded between 2016 and 2021. All reported differences reached the statistical significance. The smallest changes in body height were observed among 8-year-olds between 1986 and 1996 (0.05 cm), as well as among 13-year-olds (−1.02 cm) and 17-year-olds (0.21 cm) between 2006 and 2016.

**Figure 2:**
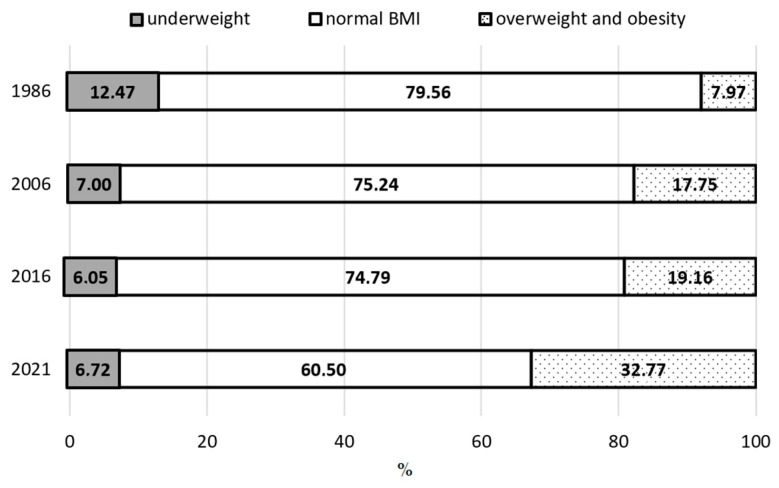
Prevalence of underweight, normal weight, and excess body weight among boys aged 13 years in 1986, 2006, 2016, and 2021.

A similar pattern of change was observed in the body mass (Table 3, Figure 2). Over the thirty-five-year observation period, the most significant and statistically meaningful increase in body mass was recorded in boys during the pubertal period, amounting to 12.54 kg. In the postpubertal and prepubertal groups, the increases were 9.68 kg and 5.90 kg, respectively. The largest and statistically significant differences in body mass among 8- and 13-year-old boys were observed during the COVID-19 pandemic period (2016–2021), with increases of 2.50 kg and 5.94 kg, respectively. In contrast, during the periods from 1986 to 1996 (for 8-year-olds: 0.10 kg) and from 2006 to 2016 (for 13-year-olds: −0.56 kg; for 17-year-olds: 0.44 kg), body mass changes remained minimal and near negligible.

Long-term changes in basic somatic characteristics were also reflected in Body Mass Index (BMI) values. Between 1986 and 2021, a systematic increase in mean BMI values was observed: 2.38 kg/m^2^ in 13-year-olds, 2.36 kg/m^2^ in 17-year-olds, and 1.50 kg/m^2^ in the youngest age group of 8-year-olds (Table 4). The most substantial increases in the BMI were recorded in the prepubertal group (0.78 kg/m^2^) and the pubertal group (1.00 kg/m^2^) during the relatively short 2016–2021 period, suggesting a possible acceleration of the nutritional transition in recent years. In the postpubertal group, the greatest rise in the BMI (1.21 kg/m^2^) was noted between 1996 and 2006. All of these changes reached statistical significance. The smallest, statistically non-significant change was observed in 8-year-olds between 1996 and 2006 (0.00 kg/m^2^). During the 2006–2016 period, a slight decrease in the BMI was recorded in both 13-year-olds (−0.03 kg/m^2^) and 17-year-olds (−0.06 kg/m^2^).

The overall picture of secular trends in the Body Mass Index (BMI) observed across the entire study population may obscure significant differences present within specific chronological age groups. Therefore, particular attention must be paid to the analysis of changes in the prevalence of underweight, normal weight, and overweight/obesity among boys aged 8, 13, and 17 years.

**Figure 3:**
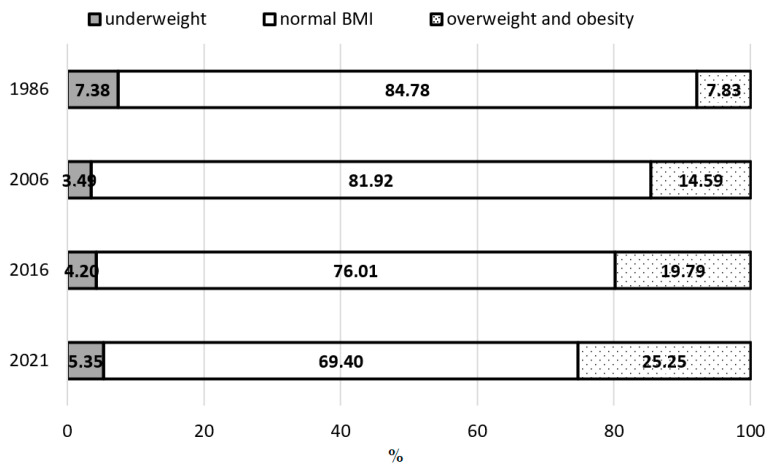
Prevalence of underweight, normal weight, and excess body weight among boys aged 17 years in 1986, 2006, 2016, and 2021.

Based on the data presented in Figure 1, Figure 2 and Figure 3 and Table 5, it was observed that between 1986 and 2021, the percentage of boys classified as underweight showed a clear downward trend, especially among adolescents aged 13 and 17. In the prepubertal group (8 years), the decrease was 1.11%; in the pubertal group (13 years) it was 5.75%; and in the postpubertal group (17 years) it was 2.03%. When analyzing changes over shorter time intervals, a varied rate of decline in the underweight prevalence was evident across age groups. The largest decreases occurred among 8-year-olds between 2016 and 2021, among 13-year-olds between 1986 and 2016, and among 17-year-olds between 1986 and 2006.

The findings also indicate a systematic decline in the proportion of boys with a normal BMI, i.e., appropriate body weight relative to height. Over the entire study period, this proportion significantly decreased across all three age groups: by 20.79% among 8-year-olds, 19.06% among 13-year-olds, and 15.38% among 17-year-olds. All differences reached statistical significance. A closer examination of individual time intervals revealed that the most marked declines in the prevalence of a normal body weight occurred among 8-year-olds from 1986 to 2016 and among 13- and 17-year-olds from 2006 to 2021.

Throughout the study period (1986–2021), a systematic increase was noted in the prevalence of excessive body weight, encompassing both overweight and obesity. This increase affected all examined age groups. In the prepubertal group (8 years), the percentage of boys with excessive body weight increased by 20.89%; by 24.80%in the pubertal group (13 years); and by 17.42% in the postpubertal group (17 years). The most dynamic changes occurred between 2006 and 2021 across all groups. Particularly concerning, however, is the marked increase in overweight and obesity observed in the most recent interval (2016–2021), despite the relatively short period between assessments. Such a sharp rise over a brief timeframe may point to a deepening public health crisis among children and adolescents, likely driven by a deteriorating lifestyle, reduced physical activity, changes in dietary habits, and the impact of environmental factors—including the consequences of the COVID-19 pandemic.

## 4. Discussion

The phenomenon of secular trends, understood as long-term changes in the physical development of populations, has long been a subject of interest for anthropologists, medical professionals, and public health specialists. These trends primarily concern increases in the average height and body weight as well as the pace of pubertal maturation. The upward trend observed since the mid-20th century has been associated with improved environmental and socioeconomic conditions, such as easier access to food, medical care, and education [22,23,24,25]. The observed changes in the somatic characteristics of boys from Eastern Poland between 1986 and 2021 also confirm the presence of secular trends. The magnitude of these changes largely depends on environmental and socioeconomic conditions, as shown by both national and international studies [26,27,28,29]. In less economically developed countries, the pace of physical growth tends to be slower, while improvements in living conditions contribute to the acceleration of physical development in children and adolescents. In highly industrialized countries, an increase in body weight is more commonly observed than in height, indicating a shift toward overweight and obesity [27,29,30].

Throughout the study period, Poland experienced a significant socioeconomic transformation. Each time point of the data collection reflected distinct contextual conditions: 1986 marked the decline of the centrally planned economy; 1996 occurred during the post-communist transition; 2006 corresponded with the EU accession; 2016 reflected economic stabilization; and 2021 coincided with the COVID-19 pandemic. These contextual shifts influenced somatic development. The greatest increase in height was observed among 13-year-olds, which may suggest accelerated biological maturation. Notable changes were observed during the prepubertal period (1996–2006) and pubertal and postpubertal periods (2016–2021), which may be partly attributable to the COVID-19 pandemic and related lifestyle disruptions; however, our study did not collect objective data on physical activity or diets, and therefore this interpretation should be treated with caution. Similar changes in body weight were noted especially between 2006 and 2021. The results presented in this study are consistent with the findings of Dobosz [31], who reported a slowdown in the height growth among children between 1999 and 2009, alongside accelerated development in younger children. More recent data from Tomaszewski et al. [32] confirm a renewed increase in height and body weight in the child and adolescent population.

Eastern Poland, which is historically less economically developed, has experienced specific developmental delays. The observed changes may partly reflect the mechanism of catch-up growth that occurs after the removal of stressors. This is supported by studies by Saczuk [9] and Kryst et al. [33], who found the greatest increases in height occurred between 1983 and 2000 and in body weight between 2000 and 2010. After 2010, growth rates slowed, possibly indicating an approach to genetically determined limits.

It should be emphasized that the study methodology remained consistent across all measurement points, ensuring the reliability of temporal comparisons. However, certain interpretative limitations exist. The absence of demographic and epidemiological data—such as the health status, migration, or the family structure—limits the understanding of how these factors affect somatic development. Moreover, individual socioeconomic variables, such as family income or parental education, were not collected in our study, which limits our ability to directly analyze their impact on the observed trends. We acknowledge that some causal interpretations regarding socioeconomic factors are based on the existing literature and broader contextual knowledge rather than our specific data. Likewise, the lack of direct lifestyle information for the study participants means that potential links between the COVID-19 pandemic and changes in weight or the BMI cannot be objectively confirmed. Additionally, the ambiguous influence of external events such as the COVID-19 pandemic requires further investigation, particularly in the context of changes in youth lifestyles and health behaviors. Despite these limitations, the findings provide important insights into the relationship between socioeconomic conditions and the development of children and adolescents in Poland.

Modern health challenges, such as the obesity epidemic, have led researchers to focus not only on height but increasingly on weight-to-height proportions. In developed countries, weight gain often outpaces height increases, contributing to a rising prevalence of overweight and obesity [26,34]. Recent Swedish studies [35,36] indicate that fluctuations in the BMI during puberty may serve as an independent risk factor for cardiovascular disease. These studies have shown a significant rise in the pubertal BMI variability over the past 45 years [36]. Incorporating such analyses into studies of Polish populations may yield valuable epidemiological insights into obesity dynamics.

In the studied population of boys from Eastern Poland, a systematic increase in BMI values was observed. This increase was primarily due to a decline in the number of children with normal weight-for-height proportions and a concurrent rise in the prevalence of overweight and obesity. These trends are consistent with findings by Tomaszewski et al. [32] and international analyses [37]. While some developed countries, such as Australia, the United States, and the United Kingdom, report the stabilization of these trends [38,39], the issue remains pressing in countries such as Germany [40], South Korea [41], and the United States [42]. In Eastern Poland, these trends have intensified particularly in the past five years, likely due to pandemic-related restrictions and broader socioeconomic changes. Hypotheses regarding the influence of social programs such as Family 500+ require further in-depth investigation, as the current evidence is inconclusive. It should be emphasized that our study lacks direct empirical data on the impact of this program on child health indicators, so this reference remains a hypothesis that warrants future research.

Although a decrease in the prevalence of underweight was also observed during the study period, this phenomenon should be interpreted with caution. A lower percentage of underweight children does not necessarily reflect an improved nutritional status but may result from a general shift in the BMI distribution toward higher values. Similar trends have been confirmed in the latest report by the NCD Risk Factor Collaboration [6]. In 2022, obesity became more prevalent than underweight among children and adolescents, clearly indicating the dominance of increasing overweight and obesity over reductions in malnutrition. Given the lack of individual socioeconomic data in our study, these interpretations should be treated with caution. We emphasize the need for future research incorporating comprehensive lifestyle and socioeconomic variables to clarify the underlying causes of observed trends. Such a general upward shift in the BMI distribution may mask hidden malnutrition issues, as a higher BMI does not always reflect a healthy body composition. Without direct body composition data, BMI trends should therefore be interpreted cautiously, since increases in the BMI may stem from rises in fat mass rather than improvements in lean mass or overall health. This underscores the limitations of using the BMI as the sole indicator of nutritional status and highlights the importance of considering the balance between lean and fat mass. In light of this, the observed decrease in the underweight prevalence among children and adolescents in Eastern Poland should not be interpreted as compensatory progress but rather as a manifestation of an overall upward shift in the BMI distribution. At the same time, the dynamic increase in overweight and obesity poses a key threat to the health of this age group. The observed secular increase in the BMI and the concomitant rise in overweight and obesity among boys from Eastern Poland have clear clinical implications. Excess adiposity during childhood and adolescence is a well-documented risk factor for the early onset of metabolic syndrome, type 2 diabetes, hypertension, and cardiovascular disease in later life [43,44]. An elevated pubertal BMI variability, as noted in recent international studies, may further compound these risks by influencing long-term cardiometabolic health trajectories [45]. These findings underscore the urgent need for pediatricians and endocrinologists to monitor BMI trends closely and to implement early interventions aimed at mitigating future clinical morbidity.

The presented results indicate that changes in the somatic characteristics of boys from Eastern Poland reflect both global secular trends and local socioeconomic conditions. There is a simultaneous acceleration of growth and a rise in overweight and obesity, occurring at the expense of children with normal weight-for-height proportions. This situation requires decisive preventive actions, focusing on the promotion of healthy lifestyles, increased physical activity, and nutritional education. Such interventions should be tailored to specific local environments, taking into account not only biological factors but also psychosocial and economic determinants. Given these findings, clinical practitioners—particularly pediatricians, primary care physicians, and endocrinologists—should incorporate regular screening for the BMI and related metabolic markers during routine health visits. The early identification of an elevated BMI or rapid pubertal BMI increases could prompt timely lifestyle interventions focusing on diet, physical activity, and behavioral modifications, consistent with current clinical guidelines [46]. Moreover, regional preventive programs tailored to the specific socioeconomic context of Eastern Poland may enhance efficacy by addressing local determinants of obesity and malnutrition.

This study contributes novel insights to the existing literature as one of the few to analyze somatic changes over a period of more than three decades (1986–2021) in Eastern Poland. It enables a comprehensive assessment of the impacts of systemic transformation, European integration, and global events such as the pandemic. In light of emerging evidence highlighting the importance of BMI variability during puberty as a predictor of later health issues, future research should consider incorporating the pubertal BMI change indicator as a supplementary analytical tool. Further studies should also include contextual data and quality-of-life indicators to allow a more comprehensive assessment of the factors shaping the development of children and adolescents. These recommendations align with contemporary clinical guidelines that emphasize the prevention of childhood obesity through multifaceted approaches involving nutritional counseling, physical activity promotion, and psychosocial support [47]. Our findings support the integration of such guidelines into public health policy and clinical practice, particularly in regions undergoing rapid socioeconomic transformation.

## 5. Conclusions

The long-term analysis of somatic development in boys from Eastern Poland between 1986 and 2021 confirms the presence of secular trends shaped by both global and local socioeconomic changes. Increases in height, body weight, and BMI reflect improved living conditions but also signal emerging health risks, particularly the rising prevalence of overweight and obesity. These changes were closely linked to major historical and socioeconomic transformations in Poland, including systemic transition, EU accession, and the COVID-19 pandemic—each influencing developmental outcomes. The decline in the underweight prevalence, although seemingly positive, likely reflects a general upward shift in the BMI distribution rather than a true improvement in nutritional status. These findings underscore the need for targeted preventive strategies focused on physical activity and nutrition, tailored to the specific needs of regional populations. From a clinical perspective, these trends highlight the urgent need for pediatricians, endocrinologists, and primary care providers to implement early screening and intervention protocols aimed at preventing obesity-related complications, such as metabolic syndrome, type 2 diabetes, and cardiovascular disease.

Future research should integrate comprehensive lifestyle and socioeconomic variables to clarify the underlying causes of the observed trends. Specifically, incorporating contextual indicators such as environmental factors (e.g., urbanization, access to recreational areas) and behavioral factors (e.g., physical activity patterns, dietary habits) is essential. Furthermore, the consideration of the pubertal maturation status and BMI variability could improve the understanding of health risk trajectories and serve as valuable predictors of long-term health outcomes in children and adolescents. Given the current study’s lack of direct lifestyle data, we emphasize the importance of including such comprehensive variables in future studies to provide a more nuanced analysis of somatic development and associated health risks. Ultimately, integrating these findings into clinical practice can support the development of effective, individualized prevention and management strategies to improve pediatric health outcomes in the region.

## Figures and Tables

**Table 1:** Number of boys examined by age group and year of measurement.

Year	Age 8	Age 13	Age 17	Total
1986	1923	2094	1189	5206
1996	236	299	146	681
2006	483	1025	883	2391
2016	457	615	475	1547
2021	2091	940	316	3347
Total	5190	4973	3009	13,172

**Table 2:** Mean body height of boys examined between 1986 and 2021 and values of ANOVA and Newman–Keuls post hoc test.

Age	Year																					
	1986 (I)					1996 (II)				2006 (III)				2016 (IV)					2021 (V)			
	*n*	Mean		SD		*n*	Mean	SD		*n*		Mean	SD	*n*		Mean		SD	*n*	Mean		SD
8	1923	127.37		5.88		236	127.42	7.18		483		130.28	5.96	457		132.46		6.04	2091	134.53		6.58
13	2094	152.96		8.43		299	156.44	8.78		1025		159.82	9.45	615		158.80		9.91	940	163.58		9.37
17	1189	173.98		6.38		146	174.94	7.17		883		175.99	8.53	475		176.20		7.31	316	178.81		6.80
**Values of ANOVA and Newman–Keuls Post Hoc Test**																						
**Age**	**ANOVA**		**I–II**		**I–III**		**I–IV**		**I–V**		**II–III**		**II–IV**		**II–V**		**III–IV**		**III–V**		**IV–V**	
8	355.63		0.16		12.93 *		22.12 *		51.24 *		8.14 *		14.22 *		23.41 *		7.55 *		19.04 *		9.06 *	
13	258.98		8.81 *		19.92 *		28.16 *		42.32 *		5.24 *		8.05 *		16.83 *		3.13 *		14.42 *		13.03 *	
17	31.53		2.12		8.77 *		7.93 *		14.79 *		2.28		2.58		7.50 *		0.72		8.34 *		6.97 *	

* Significant differences at the level of *p* ≤ 0.05.

**Table 3:** Mean body weight of boys examined between 1986 and 2021 and values of ANOVA and Newman–Keuls post hoc test.

Age	Year																					
	1986 (I)					1996 (II)				2006 (III)				2016 (IV)				2021 (V)				
	*n*	Mean		SD		*n*	Mean	SD		*n*		Mean	SD	*n*		Mean	SD	*n*		Mean		SD
8	1923	26.48		4.64		236	26.58	5.43		483		27.78	4.81	457		29.88	6.74	2091		32.38		7.79
13	2094	43.00		8.99		299	45.66	8.62		1025		50.16	10.42	607		49.60	10.43	940		55.54		12.73
17	1189	63.59		8.87		146	64.54	8.32		883		68.82	9.83	475		69.26	10.57	316		73.27		11.10
**Values of ANOVA and Newman–Keuls Post Hoc Test**																						
**Age**	**ANOVA**		**I–II**		**I–III**		**I–IV**		**I–V**		**II–III**		**II–IV**		**II–V**		**III–IV**		**III–V**		**IV–V**	
8	238.85		0.32		5.72 *		14.62 *		41.80 *		3.38 *		9.21 *		18.90 *		7.20 *		20.39 *		10.84 *	
13	270.59		5.94 *		19.76 *		25.93 *		44.09 *		7.70 *		9.45 *		20.54 *		1.51		15.75 *		16.45 *	
17	86.92		1.59		17.23 *		15.29 *		22.38 *		7.01 *		7.30 *		12.77 *		1.13		9.94 *		8.08 *	

* Significant differences at the level of *p* ≤ 0.05.

**Table 4:** BMI values of boys examined between 1986 and 2021 and values of ANOVA and Newman–Keuls post hoc test.

Age	Year																							
	1986 (I)					1996 (II)					2006 (III)				2016 (IV)					2021 (V)				
	*n*	Mean		SD		*n*	Mean		SD		*n*		Mean	SD	*n*		Mean		SD	*n*		Mean		SD
8	1923	16.24		1.98		236	16.37		1.75		483		16.37	1.78	457		16.96		3.27	2091		17.74		3.24
13	2094	18.23		2.59		299	18.66		2.11		1025		19.64	2.09	607		19.61		2.98	940		20.61		3.63
17	1189	20.97		2.41		146	21.09		1.69		883		22.30	1.90	475		22.24		2.63	316		23.33		3.22
**Values of ANOVA and Newman–Keuls Post Hoc Test**																								
**Age**	**ANOVA**		**I–II**		**I–III**			**I–IV**		**I–V**		**II–III**		**II–IV**		**II–V**		**III–IV**			**III–V**		**IV–V**	
8	89.01		1.00		1.36			7.36 *		25.27 *		-		3.92 *		10.62 *		4.81 *			14.45 *		8.04 *	
13	139.23		3.57 *		15.38 *			19.00 *		31.14 *		6.91 *		7.66 *		15.09 *		0.30			9.87		11.03 *	
17	85.69		0.81		13.89 *			17.78 *		22.14		7.22 *		8.04 *		13.29 *		0.63			8.92 *		9.33 *	

* Significant differences at the level of *p* ≤ 0.05.

**Table 5:** Changes in the prevalence of underweight, normal BMI, and excess body weight among boys assessed between 1986 and 2021.

	1986–2006	1986–2016	1986–2021	2006–2016	2006–2021	2016–2021
Age 8						
underweight	+3.74 *	+5.04 *	−1.11	+1.30	−4.85 *	−6.15 *
normal BMI	−9.63 *	−15.64 *	−20.79 *	−6.00 *	−11.16 *	−5.15 *
overweight and obesity	+5.93 *	+10.59 *	+20.89 *	+4.66 *	+15.96	+10.30 *
Age 13						
underweight	−5.47 *	−6.42 *	−5.75 *	−0.95	−0.28	+0.67
normal BMI	−4.32 *	−4.77 *	−19.06 *	−049	−14.74 *	−14.29 *
overweight and obesity	+9.78 *	+11.19 *	+24.80 *	+1.41	+15.02 *	+13.61 *
Age 17						
underweight	−3.88 *	−3.18 *	−2.03 *	+0.70	+1.85	+1.15
normal BMI	−2.86 *	−8.77 *	−15.38 *	−5.91 *	−12.52 *	−6.61 *
overweight and obesity	+6.76 *	+11.96 *	+17.42 *	+5.20 *	+10.66 *	+5.46 *

* Significant differences at the level of *p* ≤ 0.05.

## Data Availability

Dataset available on request due to restrictions.
